# Effects of IL-1β, IL-6 and IL-8 on erythrocytes, platelets and clot viscoelasticity

**DOI:** 10.1038/srep32188

**Published:** 2016-08-26

**Authors:** Janette Bester, Etheresia Pretorius

**Affiliations:** 1 Department of Physiology, University of Pretoria, South Africa

## Abstract

Complex interactions exist between cytokines, and the interleukin family plays a fundamental role in inflammation. Particularly circulating IL-1β, IL-6 and IL-8 are unregulated in systemic and chronic inflammatory conditions. Hypercoagulability is an important hallmark of inflammation, and these cytokines are critically involved in abnormal clot formation, erythrocyte pathology and platelet hyper-activation, and these three cytokines have known receptors on platelets. Although these cytokines are always unregulated in inflammation, we do not know how the individual cytokines act upon the structure of erythrocytes and platelets, and which of the viscoelastic clot parameters are changed. Here we study the effects of IL-1β, IL-6 and IL-8 at low physiological levels, representative of chronic inflammation, by using scanning electron microscopy and thromboelastography. All three interleukins caused the viscoelastic properties to display an increased hypercoagulability of whole blood and pathology of both erythrocytes and platelets. The most pronounced changes were noted where all three cytokines caused platelet hyper-activation and spreading. Erythrocyte structure was notably affected in the presence of IL-8, where the morphological changes resembled that typically seen in eryptosis (programmed cell death). We suggest that erythrocytes and platelets are particularly sensitive to cytokine presence, and that they are excellent health indicators.

Complex interactions exist between cytokines and inflammation, and specifically the interleukin family plays a fundamental role in systemic inflammation. Particularly IL-1β, IL-6 and IL-8 are present in whole blood, and measurable (in pg.mL^−1^) in most systemic inflammatory conditions. An important hallmark of systemic inflammation is a pathological coagulation potential, and hypercoagulation is also found in (most) inflammatory conditions (discussed extensively in refs 1, 2, 3, 4, 5, 6, 7, 8, 9, 10, 11, 12).

Proinflammatory cytokines are capable of activating the coagulation system and also play an important role in the down-regulation of important physiological anticoagulant pathways^13^. Also, plasma levels of several inflammation markers have been found to be associated with future cardiovascular risk in a variety of clinical settings^14^. The coagulation system is primarily triggered in response to damage to the endothelium, which allows the exposure of blood clotting factors to extravascular tissue. In healthy individuals, hemostasis is closely regulated by several anticoagulant mechanisms that balance the procoagulant forces and thus preventing untimely vascular clotting^15^. Pro-inflammatory cytokines and chemokines can affect all coagulation pathways^16^. Therefore the intricate relationship between the presence of cytokines resulting in inflammation and hyper-coagulation, are particularly relevant in the pathogenesis of vascular disease. See a high-level overview (Fig. 1) that illustrates how coagulation is affected by inflammation and a changed cytokine profile; adapted from refs 17, 18, 19, 20, 21.

In this paper, we are particularly interested in the effects of circulating IL-1β, IL-6 and IL-8 on both erythrocytes (RBCs) and platelets, and how exposure to these interleukins may affect platelet and RBC structure during inflammation.

Interleukin 1 Receptor 1 (IL1R1) and its ligand, IL1β, are unregulated in cardiovascular disease and infection^22^. IL-1β is also known to be present in autoimmune conditions and contributes to several chronic diseases, including atherosclerosis and type 2 diabetes^23^,^24^,^25^. IL-1α and IL-1β have a natural antagonist, IL-1Ra and both bind to the same receptor molecule, IL-1 receptor 1; see refs 26,27 for extensive discussion on the role of particularly IL-1β in inflammation. Bursts of IL-1β are involved in acute attacks of systemic or local inflammation, and also in myocardial infarction or stroke^23^. IL-1β also plays a significant role in the inflammation induced by *Helicobacter pylori*^28^. RBCs do not have an IL-1β receptor binding site, but platelets express the IL1R1 receptor, and respond to IL-1β; and in e.g. maturing thrombi platelets can accumulate IL-1β^29^,^30^.

IL-6 is a multifunctional cytokine that regulates the immune response, haemopoiesis, the acute phase response, inflammation^31^ and the central nervous system^31^,^32^. Its expression is high and transiently unregulated in nearly all pathophysiological inflammatory conditions and also in autoimmune diseases^33^,^34^. IL-6 trans-signaling is also critically involved in the maintenance of a disease state, by promoting transition from acute to chronic inflammation^35^. IL-6 exerts its biological activities through two molecules: IL-6R (IL-6 receptor) and by the membrane-bound β-receptor glycoprotein 130 (gp130)^31^,^36^. Transduction of the signal is mediated by gp130 and also by trans-signaling, where IL-6 binds to soluble forms of the IL-6R (sIL-6R). These agonistic IL-6/sIL-6R complexes can in principle activate all cells due to the uniform expression of gp130 (which is present on all cells)^33^,^34^,^37^,^38^. Resting platelets also express gp130 on their membranes, and in the presence of IL-6 (produced by stressed endothelial cells), platelet-derived IL-6 trans-signaling happens, and could be crucial in the development of inflammation within a damaged vessel^39^ and in platelet thrombogenicity^40^.

IL-8 is also a well-known circulating inflammatory cytokine^41^,^42^. Macrophages and other cell types such as epithelial cells, airway smooth muscle cells and endothelial cells produce IL-8. There are many receptors on the surface membrane capable of binding IL-8; the most frequently studied types are the G protein-coupled serpentine receptors CXCR1 and CXCR2^43^,^44^. Platelets may have IL-8 receptors, and it was found that IL-8–dependent activation of washed platelets may happen, leading to procoagulant activity^45^.

Plasma and serum levels of IL-1β, IL-6 and IL-8 are measured in pg.mL^−1^ and the mean concentrations of healthy individuals and those with inflammatory conditions are typically as follows:

- IL-1β, ±0.7–1.1 pg.mL^−1^ in healthy individuals and^46^ and ±30 pg.mL^−1^ in unstable angina pectoralis^14^.
- IL-6: ±4 pg.mL^−1^ in healthy individuals and levels in psoriasis ±14 pg.ml^−1^ 47.
- IL-8: ±14 pg.mL^−1^ in healthy individuals and in patients with active psoriasis: ±40 pg.mL^−1^ 47 and unstable angina pectoralis^14^.

After taking all the evidence into consideration, we note that there is no research that we could find, that specifically looks at the individual effects of the 3 cytokines on RBCs and platelets, to determine their individual effects, using specifically ultrastructure. The question also arose: are all cytokines equal or is there 1 signal one among the 3 that has a more pronounced effect than the others. Our hypothesis therefore is that IL-1β, IL-6 and IL-8 individually cause changes to the coagulation profiles and to platelets due to their binding to platelets; but that IL-6 might possibly show the most effects on RBCs, due to the fact that it has an universal binding site on all cells, including RBCs. We tested this hypothesis by looking at clotting, platelet hyper-coagulation and eryptosis, adding these cytokines individually, at low concentrations, and used thromboelastography and scanning electron microscopy to test our hypothesis. We also compared the results from this study with ultrastructural research previously done using blood from inflammatory conditions, with known cytokine upregulation.

## Materials and Methods

### Ethical statement

This study was approved by the Ethical Committee of the University of Pretoria (South Africa). A written form of informed consent was obtained from all healthy donors (available on request). The methods were carried out in accordance with the approved guidelines. Blood was collected and methods were carried out in accordance with the relevant guidelines of the ethics committee (ethics number: 506/2014 and 298/2016: E Pretorius and J Bester: principal investigators for use of control blood; ethics number for Alzheimer type study: 81/2013; ethics number for Parkinson’s disease study: 80/2013; ethics number for Type II study: 68/2014; and ethics number for rheumatoid arthritis study: 462/2013). We adhered strictly to the Declaration of Helsinki.

### Concentration of interleukins used

We exposed healthy whole blood to a final exposure concentration of 20 pg.mL^−1^ IL-1β, 15 pg.mL^−1^ IL-6 and 40 pg.mL^−1^ IL-8, for 10 minutes at room temperature, as these concentrations are in line with concentrations of these cytokines found in plasma and serum of systemic chronic inflammation^14^,^46^,^47^. Concentrations of cytokines are mostly measured in either plasma or serum. We took exceptional care to determine from literature the concentrations in healthy and diseased individuals. We then decided on concentrations that are low, even for systemic inflammation (but higher than in healthy individuals); and not nearly as high as is found in acute inflammation. The interleukins were purchased from Sigma (catalogue numbers: IL-1β: I9401, IL-8: I1645, IL-6: I2786).

### Healthy volunteer details and blood collection

Blood samples were obtained from 10 healthy individuals of ages ranging from 18 to 60. Blood was collected in one 4.5 mL citrate tube. This collection was done by a medical doctor and all handling of samples were performed under very strict aseptically conditions, in order to prevent contamination of samples.

### Inflammatory patient details and blood collection

In this paper, we include SEM micrographs taken as part of previous published papers, to support the *in vitro* results presented here^48^,^49^,^50^,^51^,^52^. The inflammatory blood sample preparation was done as discussed for the healthy volunteers and also in the various papers itself.

### Data sharing

Raw data, extensive SOPs for TEG and SEM, including original images without color and micrographs can be accessed at: https://1drv.ms/f/s!AgoCOmY3bkKHbAm9Z0xJbqtthTA, and on the corrresponding author’s researchgate profile, https://www.researchgate.net/profile/Etheresia_Pretorius, as raw data.

### Thromboelastography

Coagulation parameters, using whole blood (WB) of healthy individuals, were done using thromboelastography (TEG). WB collected in citrate tubes were left for 30 minutes at room temperature before the experiment was started. 30 minutes after blood was drawn in citrated tubes, the WB was incubated for 10 minutes with each of the interleukins at the final exposure concentration mentioned above. 340 μl of the interleukin-incubated WB and naïve WB were placed in a disposable cup in a computer-controlled TEG hemostasis system (Model 5000, Hemoscope, Niles, IL), with addition of 20 μl CaCl_2_ as the last step to initiate clotting. Thrombelastographic data was collected until maximum amplitude (MA) is reached or 60 min had elapsed^53^,^54^,^55^,^56^,^57^,^58^. See Table 1 for the parameters that are obtained when whole blood-clotting profiles are studied using the TEG; this table was adapted from refs 59, 60, 61. TEG is typically used to determine clot formation and clot strength^62^. Statistical analysis was done with the program StatsDirect and p-values were obtained using non-parametric Mann-Whitey analysis.

### Scanning electron microscopy

After the blood was collected (also left in citrate tubes fro 30 minutes) and incubated with the interleukins for 10 minutes, 10 μl of WB with added interleukins and naïve WB were placed directly on a glass cover slip, fixed, dehydrated, dried, mounted and coated with carbon according to previously described methods^63^. A high-resolution crossbeam 540 Zeiss scanning electron microscope was used to study the surface morphology of erythrocytes and platelets. Micrographs were taken at 1 kV. Due to the high quality of the SEM images, no processing was done except to add color using Adobe®Photoshop CS6® version 13.0 × 64. For a significant selection of our raw data see https://1drv.ms/f/s!AgoCOmY3bkKHbAm9Z0xJbqtthTA.

To relate our *in vitro* results to actual *in vivo* results as seen in whole blood of actual patients with known systemic inflammation, we add SEM micrographs from individuals with type II diabetes, rheumatoid arthritis, Parkinson’s disease and Alzheimer’s type dementia.

## Results

Table 2 shows the TEG whole blood results of the samples. Addition of the three cytokines to whole blood, all showed an increased clotting potential, confirming their role in increased hypercoagulability, during inflammation. Noteworthy changes in the following parameters are that the R-time was shortened with addition of all three interleukins, with a decreased maximum velocity of clot growth (MA), as well as a decreased velocity of clot growth (MRTG) and a decrease in time before maximum velocity of clot growth (TMRTG) and clot strength (TTG). Significant changes in MRTG, TMRTG and TTG are indicative of specific modifications during fibrin formation from fibrinogen, while the other parameters are indicative of interactions of all cellular components involved in coagulation. From these results, IL-1β caused significant changes in the blood clotting profiles (see Table 2). The resulting clot is formed faster, is significantly less stable (MA), with a significantly decreased velocity (MRTG) to reach the maximum clot growth, resulting in a unstable and fragile clot. This is an indication of clot hypercoagulability due to IL-1β exposure. IL-6 also caused the clot to form faster, it is also significantly less stable, but it has a significantly decreased TMRTG and TTG, indicating that a unstable, fragile clot is formed faster. Il-6 therefore caused a more hypercoagulable clot than IL-1β, likely due to a significant change to fibrin(ogen). IL-8 caused significant changes in all TEG parameters, suggesting that it is the most potent procoagulant cytokine from the 3 studied. It caused the clot to form faster, reaching clot strengh significanlty slower, with a decreased thrombin burst, resulting in a slower fibirn cross-linking (indicated by the angle parameter). All of the above-mentioned parameters contributes to a significantly unstable clot (shown by a significantly decreased MA). IL-8 is therefore the cytokine that caused the most significant changes at all levels of coagulation, including fibrin(ogen), thrombin and cellular interactions.

Figure 2A,B shows representative RBCs and a close-up of the membrane (120 000x machine magnification) of a healthy individual is shown in Fig. 2C. Figure 2D shows a typical platelet, with slight pseudopodia formation due to contact activation. Figures 3, 4, 5 shows similar micrographs where the three interleukins were added to whole blood respectively.

It is also well-known that a general hypercoagulable state is present in various inflammatory conditions and we and others have shown changes to RBC (eryptosis) and platelets hyper-activation in various conditions, including type 2 diabetes, Parkinson’s disease, Alzheimer’s disease and rheumatoid arthritis^48^,^49^,^50^,^51^,^52^. In all of these diseases, the 3 cytokines of interest in this paper, feature prominently as major mechanistic role players in the inflammatory profiles of the diseases. See Fig. 6 to show micrographs that were taken as part of the previously mentioned studies, to support the evidence presented here. Obviously in these *in vivo* situations, all 3 cytokines are upregulated simultaneously, and in this paper the idea is to show the effects of the individual cytokines.

As seen with the TEG results, the SEM confirms that all 3 cytokines act upon the cellular component of WB, and causes platelet hyper-activation and specifically IL-8 causes changes that we relate to the initiation of RBC eryptosis and visible structural changes to RBC membranes were noted (see Fig. 5).

## Discussion

**Figure 1:**
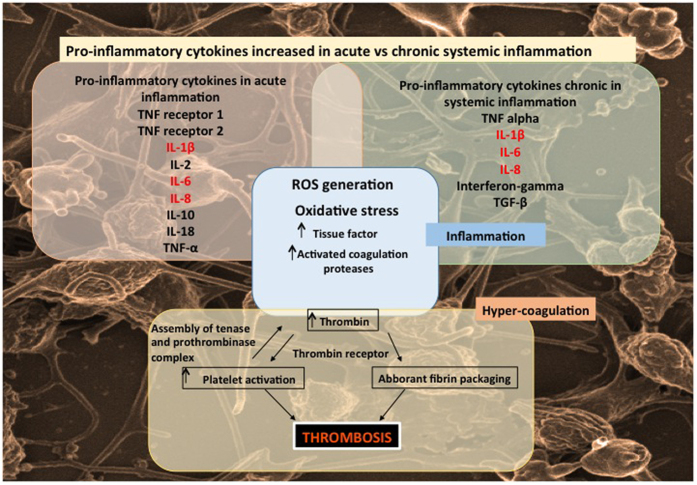
The intricate relationship between inflammation and hyper-coagulation. This diagram focuses on the bidirectional relationship between inflammation and coagulation and the role that increased pro-inflammatory cytokines in both acute and chronic systemic inflammation plays in the activation of the coagulation system.

**Figure 2:**
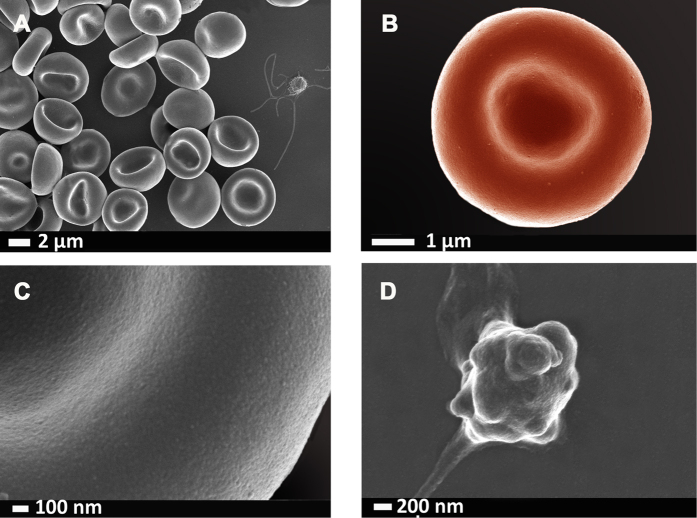
(**A**) A low magnification to show overall view with RBCs and a platelet with slight pseudopodia formation due to contact activation. (**B)** Representative RBC from a healthy individual; (**C)** high magnification of RBC membrane; (**D)** platelet showing slight pseudopodia formation due to contact activation. Micrographs were taken at 1 kV using a crossbeam 540 Zeiss scanning electron microscope. No changes were done on actual figures and color enhancement was done using Adobe®Photoshop CS6® version 13.0 × 64.

Increased levels of IL-1β, IL-6 and IL-8 are known to play an important role in both acute and chronic inflammation, with resulting pathological clotting. However, we know little about the effects of these interleukins on the ultrastructure of RBCs and platelets or how they change the individual viscoelastic properties involved in clot formation. As shown in Fig. 1, the interactions of the 3 cytokines are complex and IL-1β, IL-6 as well as IL-8, and their mechanism of action are typically implicated in pro-inflammatory induced coagulation^17^,^18^,^19^,^20^,^21^. IL-6 is primarily involved in the up-regulation of tissue factor that brings about initiation of coagulation. IL-1β down-regulates thrombomodulin and consequently causes defects in anticoagulant proteins, specifically impairing activation of protein C. The activation of protein C (in a healthy individual) is an important step in the anti-coagulant pathway. During IL-1β presence, as seen in inflammation, it acts as a procoagulant to impair protein C activity. In a healthy individual, thrombomodulin has an anti-inflammatory activity, and down-regulation due to IL-1β, impairs this protective effect. IL-8 promotes procoagulant activity, by triggering platelet activation^45^. In various papers, others and we have shown the effect of an upregulated inflammatory profile due to conditions like Alzheimer’s disease, Parkinson’s disease, T2D and rheumathoid arthritis in blood of these patients. Various research papers that support the effects of these upregulated cytokines resulting in a dysregulated immune system, specifically resulting in eryptosis, is therefore important to note^64^,^65^,^66^,^67^,^68^,^69^,^70^,^71^,^72^.

In this paper we used TEG to track clot formations over time and we used the standard procedure where we measured clotting parameters, from clot initiation until maximum clot strength or stiffness of clot were reached (MA), after addition of CaCl_2_ to reverse the anti-clotting action of citrate. TEG therefore provides descriptive clotting parameters over time. SEM ultrastructure, displays a single snapshot after 10 minutes exposure of WB to the 3 interleukins, to visually show how the interleukins affect cell structure.

**Figure 3:**
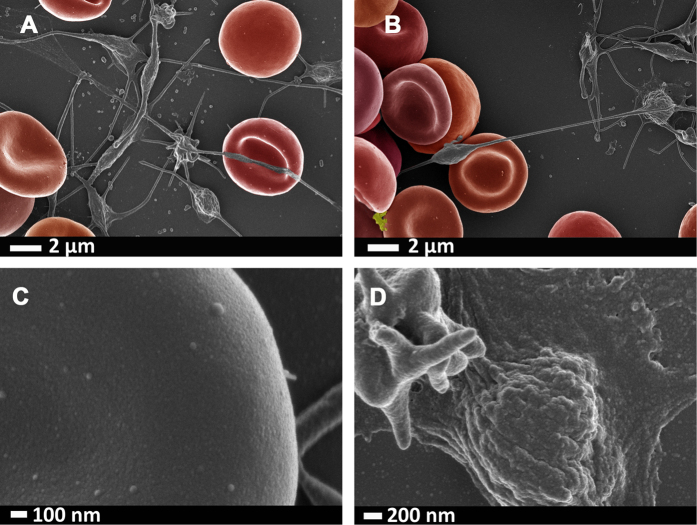
(**A**,**B)** Representative RBCs and platelet clumps from a healthy individual, after whole blood was exposed to IL-1β; (**C)** high magnification of RBC membrane; (**D)** Platelet that shows spreading and hyper-activation. Micrographs were taken at 1 kV using a crossbeam 540 Zeiss scanning electron microscope. No changes were done on actual figures and color enhancement was done using Adobe®Photoshop CS6® version 13.0 × 64.

**Figure 4:**
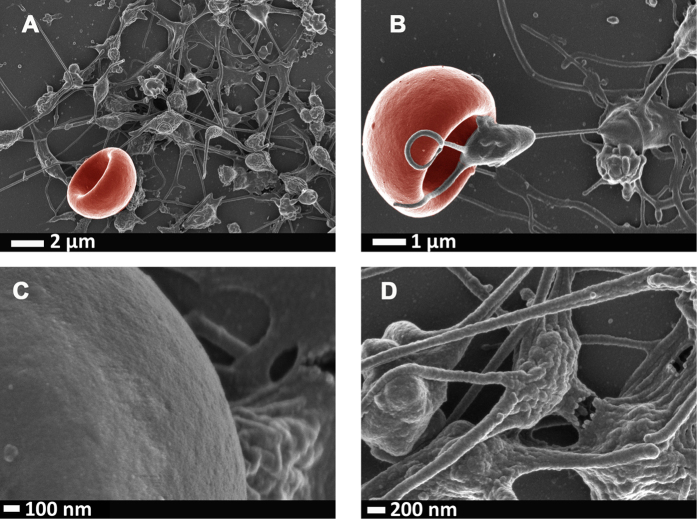
(**A**,**B)** Representative RBCs and platelet clumps from a healthy individual, after whole blood was exposed to IL-6; (**C)** high magnification of RBC membrane; (**D)** Platelet that shows spreading and hyper-activation. Micrographs were taken at 1 kV using a crossbeam 540 Zeiss scanning electron microscope. No changes were done on actual figures and color enhancement was done using Adobe®Photoshop CS6® version 13.0 × 64.

**Figure 5:**
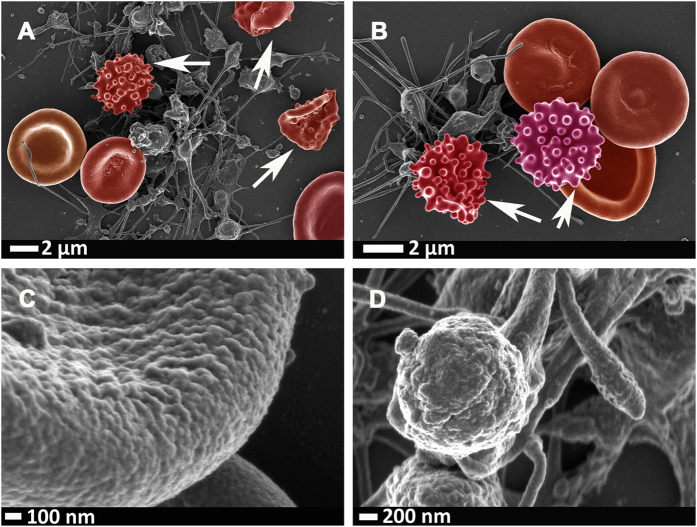
(**A**,**B)** Representative RBCs and platelet clumps from a healthy individual, after whole blood was exposed to IL-8; eryptotic cells indicated with arrows. **(C)** High magnification of RBC membrane, showing ultrastructural changes; (**D)** Platelet that shows hyper-activation. Micrographs were taken at 1 kV using a crossbeam 540 Zeiss scanning electron microscope. No changes were done on actual figures and color enhancement was done using Adobe®Photoshop CS6® version 13.0 × 64.

**Figure 6:**
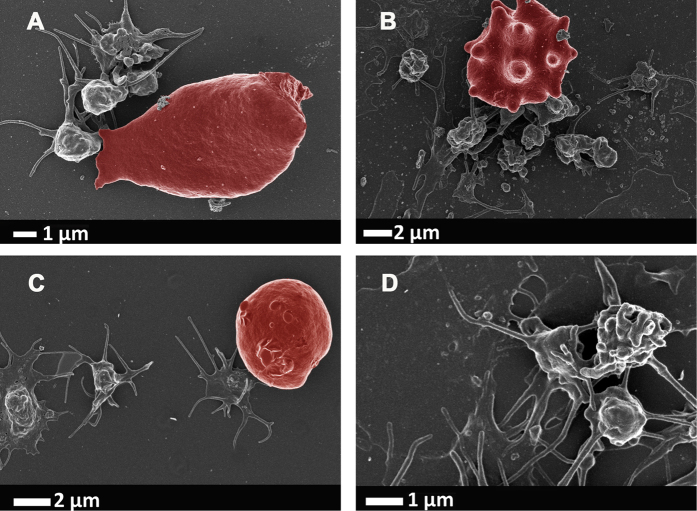
Micrographs from inflammatory diseases where IL-1β, IL-6 and IL-8 upregulation plays a fundamental role in the pathogenesis and hypercoagulability of the diseases. (**A)** Alzheimer’s disease; (**B)** Parkinson’s disease; (**C)** type 2 diabetes; (**D)** rheumathoid arthritis. Micrographs were taken at 1 kV using a crossbeam 540 Zeiss scanning electron microscope. No changes were done on actual figures and color enhancement was done using Adobe®Photoshop CS6® version 13.0 × 64.

Our hypothesis stated that IL-1β, IL-6 and IL-8 individually will cause changes to the coagulation profiles and to platelets due to their binding to platelets; but that IL-6 might possibly show the most effects on RBCs, due to the fact that it has an universal binding site on all cells, including RBCs. All 3 cytokines did indeed change clotting profiles and caused platelet hyper-activation. Here we confirmed a changed clotting profile in the presence of IL-1β, IL-6 and IL-8, using TEG. IL-8 showed the most pronounced hypercoagulability in TEG, where all the clotting parameters where significantly different to the naïve whole blood parameters (Table 2). The very sensitive SEM analysis showed changes in RBCs and platelets after exposure to all three interleukins. SEM analysis showed that, with the addition of IL-1β, IL-6 and IL-8 to whole blood (Figs 3, 4, 5), platelets were hyper-activated, showing pronounced spreading, and clumped together. They were also closely associated and attached their pseudopodia onto RBC membranes. Platelets have receptors for all three interleukins, however, IL-6, is the only one of the three that actually has a receptor on RBCs; as in principle it activates all cells due to the uniform expression of gp130. To our surprise and against our hypothesis (where we believed that possibly only IL-6 might cause eryptosis), the addition of IL-8 not only showed the most pronounced effects on TEG parameters, but also on SEM, and it caused some RBCs to become eryptotic (see Fig. 5A,B). The other 2 cytokines did not significantly affect RBC structure. Most RBC membranes, in the presence of IL-8, showed pathological membrane changes (see Fig. 5C). Eryptosis is RBC programmed cell death and has been discussed numerous times by^73^,^74^,^75^,^76^,^77^,^78^,^79^,^80^,^81^,^82^. It is characterized by cell shrinkage, cell membrane blebbing and cell membrane phospholipid scrambling^83^; and it involves COX, PGE_2_, ceramide, and activation of calpain. All of these biochemical changes inside the cell lead to the morphological pathology, which is characteristic of eryptosis. These pathologies are visible as cell shrinkage, membrane scrambling and membrane blebbing^52^,^82^. We have previously reported eryptotic RBCs in inflammatory conditions like Parkinson’s disease^52^ and Alzheimer’s disease^84^, where IL-8 is also known to be unregulated. We show micrographs from such individuals in Fig. 6. Although our results showed that IL-8 causes the most pronounced changes in WB, we could not find literature evidence that IL-8 has receptors on RBC membranes. We could also not find evidence that previous researchers have found that IL-8 is associated with the induction of eryptosis, however, it is a well-known activator of apoptosis (which is similar to eryptosis vs. programmed cell death)^85^,^86^. It is also well known that IL-8 is closely involved in the traditional apoptotic pathways where e.g. COX and PGE_2_ play important roles in apoptosis initiation. Although we do not have evidence that IL-8 indeed binds to an RBC receptor, we show here that it does indeed causes the induction of eryptosis, confirmed by morphological changes.

RBCs and platelets are particularly sensitive to systemic inflammatory changes. Ultrastructure of RBCs, platelets as well as viscoelastic properties of clots formed with whole blood, might give great insight on effects that cytokines may have on the development of conditions like atherosclerosis, thrombosis and hypercoagulability. These biophysical readouts are therefore excellent health indicators and may be exploited in therapeutic studies.

## Additional Information

**How to cite this article**: Bester, J. and Pretorius, E. Effects of IL-1β, IL-6 and IL-8 on erythrocytes, platelets and clot viscoelasticity. *Sci. Rep.* **6**, 32188; doi: 10.1038/srep32188 (2016).

## Figures and Tables

**Table 1:** TEG parameters typically generated for whole blood.

THROMBOELASTIC PARAMETERS	
R value: reaction time measured in minutes	Time of latency from start of test to initial fibrin formation (amplitude of 2 mm); i.e. initiation time
K: kinetics measured in minutes	Time taken to achieve a certain level of clot strength (amplitude of 20 mm); i.e. amplification
Α (Alpha): Angle (slope between the traces represented by R and K) Angle is measured in degrees	The angle measures the speed at which fibrin build up and cross linking takes place, hence assesses the rate of clot formation; i.e. thrombin burst
MA: Maximal Amplitude measured in mm	Maximum strength/stiffness of clot. Reflects the ultimate strength of the fibrin clot, i.e. overall stability of the clot
Maximum rate of thrombus generation (MRTG) measured in Dyn.cm^−2^.s^−1^	The maximum velocity of clot growth observed or maximum rate of thrombus generation using G, where G is the elastic modulus strength of the thrombus in dynes per cm^−2^
Time to maximum rate of thrombus generation (TMRTG) measured in minutes	The time interval observed before the maximum speed of the clot growth
Total thrombus generation (TTG) measured in Dyn.cm^−2^	The clot strength: the amount of total resistance (to movement of the cup and pin) generated during clot formation. This is the total area under the velocity curve during clot growth, representing the amount of clot strength generated during clot growth

**Table 2:** TEG results of naïve whole blood with and without added interleukins, showing medians, standard deviations and P-values (2-sided P-value taken) (Mann-whitney analysis); significance indicated in red.

TEG Results of Naïve Whole Blood With and Without Added Interleukins							
	R	K	Angle	MA	MTRG	TMRTG	TTG
Healthy individuals	8.95 (±1.9)	3.6 (±0.98)	47.8 (±7.6)	54.4 (±4.1)	4.0 (±1.4)	12.96 (±3.2)	595.9 (±114.2)
With added IL-1β	7.6 (±3.0)	4.1 (±1.2)	40.1 (±7.0)	46.6 (±6.1)	2.7 (±1.1)	10.9 (±3.7)	436.9 (±108.4)
P-value	**0.36**	**0.10**	**0.06**	***0.002***	***0.04***	**0.18**	***0.002***
With added IL-6	7.3 (±2.5)	3.4 (±1.03)	48.6 (±9.9)	50.3 (±3.2)	3.4 (±0.9)	10.5 (±3.8)	508.4 (±75.4)
P-value	**0.16**	**0.80**	**0.85**	***0.03***	**0.30**	***0.045***	***0.03***
With added IL-8	5.2 (±0.78)	8.3 (±3.4)	31 (±6.4)	31 (±4.99)	1.3 (±0.4)	6.4 (±2.1)	227.6 (±49.9)
P-value	***0.0014***	***<0.0001***	***0.0002***	***<0.0001***	***<0.0001***	***0.0020***	***<0.0001***
